# The Use of Raman Spectroscopy to Monitor Changes in the Intensity of Ratio of Integral Unsaturated Bands in Bio-Greases

**DOI:** 10.3390/molecules28073033

**Published:** 2023-03-29

**Authors:** Jolanta Drabik, Bernadetta Kaźmierczak, Rafał Kozdrach, Elżbieta Rogoś

**Affiliations:** Bioeconomy and Ecoinnovation Centre, Proecological Technologies Research Group, ŁUKASIEWICZ Research Network—Institute for Sustainable Technologies, Pułaskiego 6/10, 26-600 Radom, Poland

**Keywords:** bio-greases, *Crambe Abyssinic oil*, thermal-oxidation test, mechanical test resistance to oxidation

## Abstract

Bio-greases were developed on the basis of vegetable oil obtained from *Crambe Abyssinic* seeds. An important aspect of this research is to monitor changes in their quality taking place under the influence of external factors. Raman spectroscopy was used to identify changes taking place in the bio-lubricant under the influence of mechanical and thermal forces. The performed tests reflected the operating temperature and friction load that may occur during actual operating conditions for the lubricated friction systems. The Raman spectra provided information on qualitative changes in the structure of the tested bio-lubricants at the molecular level. The integral intensity of the bands used to assess the degree of lipid unsaturation was adopted as the evaluation criterion. The influence of the oxidation process under the PetroOxy and wear test conditions on changes in the structure of the bio-lubricants was assessed. Variation in the integral intensity of the bands (I_1655_/I_1440_) proves that the structure of vegetable lubricants changes under the influence of the tests performed. Thermal and mechanical forces influence, the bands originating in unsaturated and result in a decrease in the oxidation resistance of vegetable lubricants.

## 1. Introduction

The lubricant is an integral and inseparable element of the tribological connection and is largely responsible for proper and trouble-free operation. High temperatures and ubiquitous oxygen are two of the most important factors contributing to the ageing of lubricants formulated with vegetable oil bases. In addition, during friction of the cooperating elements of the tribological system, significant changes occur in the structure of materials caused by the operating conditions. The mechanical and physicochemical phenomena occurring on the surface of the friction node elements under the influence of temperature and pressure shape the surface layer, which changes during operation and under the influence of lubricants. During friction carried out in the open air, the lubricant degrades under the influence of oxygen and the catalytic effect of the metal from the friction surface on the lubricant oxidation process. The wide use of natural oils in lubrication technology is conditioned by their resistance to the oxidation process, which results from the presence of unsaturated bonds, as these oils contain monounsaturated and polyunsaturated fatty acids [1,2,3].

In general, despite the content of natural antioxidants in their composition, vegetable oils are characterised by insufficient, long-term oxidative stability. This limitation results from the significant content of unsaturated acids with one, two or three double bonds in the structure of vegetable oils, which are unstable and easily oxidised [1,2]. In this context, it is important to develop quick methods for identifying changes in the structure of plant lubricants under the influence of excitation. The Raman method has the best prediction ability for unsaturated fatty acid, and in particular trans unsaturated fatty acid. The differences in reactivity between oils depend on the number of double bonds, their mutual position and the geometric structure of the chain. The rate of oxidation depends on the presence of antioxidants in oils, such as tocopherols, carotenoids, phenolic compounds, and external conditions, with the most important influence being temperature, light and oxygen [2,4,5,6].

The literature reports indicate a wide spectrum of possibilities in using Raman spectroscopy for the analysis of fatty acids, lipids or proteins [7]. Philippidis et al. [8] successfully used Raman spectroscopy as a fast and non-invasive method to detect the content of vegetable oil admixtures in olive oil. Aviles et al. [9] describes the results of research on improving the tribological properties of lubricants through the use of ecological additives derived from fatty acids. Raman spectroscopy was used to analyse the traces of wear of test elements after the lubricant tribological tests. The analysis of the spectra showed that the tested additives reduce the intensity of the lubricant oxidation process that occurs during friction. Chen et al. [10] describes the analysis of friction traces using Raman spektrometry. Examined effectiveness of the use of two types of graphene as lubricant additives in polyalphaolefins (PAO) was tested. In both cases, iron oxides were present in the traces of friction, which indicates the accumulation of wear products in the rubbing elements. Song et al. [11] presents the results of research on the tribological properties of carbon materials of different morphology, used as lubricating additives in sunflower oil. The research included, inter alia, identification of oxygen-containing groups in oil and additives by means of FTIR infrared spectroscopy, and examination of lubricating dispersions and friction surfaces by Raman spectroscopy. The interaction of the Van der Waals forces between the oxygen-containing groups present in the oil and the additives had an effect on the stability of the lubricating dispersions. The analysis of Raman spectra of the lubricating dispersions and traces of friction showed the presence of the additives used in the traces of friction. Raman spectroscopy with surface enhancement (SERS) was used to study the traces of friction on silver coated balls, lubricated with vegetable oils. It was shown that during friction, fatty acids were released from the triglyceride structure of vegetable oils and adsorbed on the surface of the spheres. The fatty acid chains of unrefined rapeseed oil were more disordered than those of refined oil [12].

Raman spectroscopy is a widely used analytical technique to study, among others, the structural properties of polymer solutions [13], yielding interesting information about structural changes at different temperatures. The changes in the chemical structure of colloidal systems [14], as well as paraffin waxes and polymer microemulsions at variable temperatures were also studied [15]. Research was additionally conducted on phase transitions in hydrocarbons [16] and the assessment of the degree of crystallinity of polymer mixtures under the influence of heating [17]. Changes in the chemical structure of aqueous polymer solutions, oil bases and lubricants under the influence of various operating conditions were also assessed [18]. The publication describes the latest achievements in the analysis of edible oils using various instrumental techniques [19]. Campton et al. [20] Raman spectroscopy as a fast method for differentiating chemical structure of ferulic acid and its derivatives was indicated. Jamrógiewicz et al. [21] presents an evaluation of vibrational spectroscopy methods, such as mid-infrared, near-infrared and Raman spectroscopy (FTIR, FT-NIR, Raman) for the identification of pseudopolymorphic forms of a model active pharmaceutical ingredient. In the Raman spectra, in depending on the forming method of emulsions were observed the changes in amorphous structures, what were correlated with the results of the DWS microrheology [22]. Kuhar et al. [23] discusses the importance of Raman spectroscopy for the evaluation of protein structure and conformational changes. Changes in the molecular structure of linoleic acid under the influence of elevated temperature and UV radiation were examined using ATR-IR reflectance infrared spectroscopy and NIR vibrational spectroscopy [24]. In order to assess susceptibility to oxidation and changes in the structure of arachidonic acid under the influence of daylight and temperature, ATR-FTIR reflection spectroscopy was used [25].

The oxidation process of lubricating oil based on palm oil was observed via among others, Fourier transform infrared spectroscopy with FTIR. It was possible to study the dynamics of the appearance of oxidation products in base oil and its mixtures with additives. The effectiveness of zeolite nanoparticles containing various cations as inhibitors of lubricating oil oxidation was assessed [26]. Long et al. [27], FTIR infrared spectrometry was used to assess the chemical composition of castor oil and other plant products (glycerol, erucic acid and oleic acid) used to lubricate the DLC/Si_4_N_4_ interface. The influence of the DLC structure on the efficiency of contact lubrication with plant products was investigated. It was found that the chemical degradation process of castor oil is favoured by the amount of hybridised sp^2^ carbon atoms in the amorphous carbon structure. It has been shown that the presence of the –OH group in lubricating products reduces the coefficient of friction. Yuan et al. [28] chemical structure of vegetable oils was assessed using Fourier Transform Infrared IR (FTIR) spectroscopy, near visible infrared spectroscopy (Vis-NIR) and EEM emission fluorescence. The results of comparative studies of vegetable oils using spectral methods confirmed that the most information about the chemical structure of oils is provided by the following techniques: FTIR and Vis-NIR. Drabik et al. [29] are presented the results of research on the correlation between the content of carboxylate ions determined by infrared spectroscopy (FT-IR) and the stability of the structure of lubricants assessed on the basis of changes in penetration and lubricating properties are presented. Borin et al. [30] describes the results of research on the use of mid-infrared spectroscopy for the analysis of impurities in lubricating oil. The studies confirmed that mid-infrared spectroscopy is a suitable technique for the quantitative determination of the most important contaminants in lubricating oils. Hanifpour et al. [31] describes the results of research on the development and production of greases based on saturated PAO in situ in a single-stage manner. NMR spectroscopy was used to analyse the obtained lubricants.

In the literature on the subject of the Raman spectroscopy scattering and infrared absorption spectroscopy, they are widely used to control and evaluate the analytical purity of materials, admixtures analyzes in pure raw materials, polymerization degree or the chemical structure of obtained products or changes occurring under the influence of aging. However, the number of reports on comprehensive research on changes in the greases structure and affecting the change in functional parameters is small. This situation results, among others, from the fact that greases are characterized by much more complex features, and their structure is much easier to change compared to liquid lubricants. Knowledge about the relationship between the structure and the properties of grease as well as the thickener and base oil, and the durability of the grease is still negligible. The development of innovative plant greases is an important issue, especially from the point of view of ensuring the required functionality throughout the life of mechanical elements cooperating with friction. Supplementing information on the impact of aging factors on a change in the quality of plant greases is an important aspect of ensuring the required lubrication.

This work aims to explain the changes occurring in the chemical structure of plant greases upon thermo-oxidative ageing and mechanical loads in model tests, which have a significant impact on their resistance bio- greases to the oxidation process. Raman spectroscopy was used to monitor changes grease developed from *Crambe Abyssinic* vegetable oil under the influence of mechanical and thermal inputs carried out in model tests. Using the unsaturated and saturated bands present in vegetable oil, the change in the degree of unsaturation under the influence of thermo-oxidation (PetroOxy test) and friction node load in an one-hour anti-wear test (G_oz_) was adopted as the evaluation criterion. An important functional parameter of plant greases is their resistance to the oxidation process. A thermal analysis was proposed to control the changes of this indicator under the influence of the for extortion used. DSC (Differential Scanning Calorimetry) thermal analysis was used to assess resistance to the oxidation process of vegetable lubricants. The oxidation start temperature was taken as the index.

## 2. Results and Discussion

**Figure 1:**
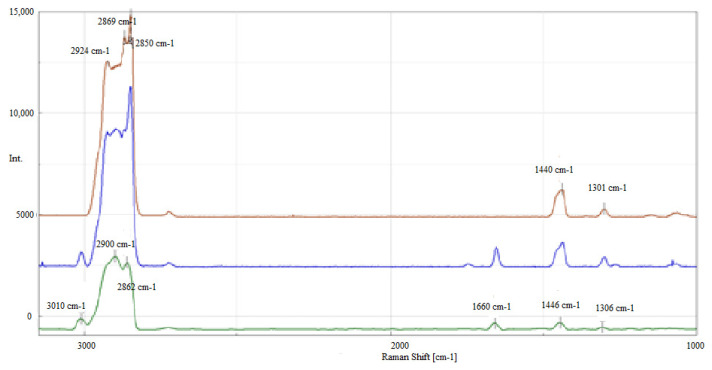
The Raman spectra of vegetable Oil A—green line, vegetable–mineral Oil AF—blue line and mineral Oil F—brown line, in the 3700–1000 cm^−1^ range.

Figure 1 shows the Raman spectra of oil A, obtained in the process of blending vegetable oil with mineral oil AF, and mineral oil F for comparison.

In Oils A and AF, bands located at 3008 cm^−1^ and 1655 cm^−1^ were identified, originating from unsaturated bonds occurring in vegetable oil (Figure 1). The characteristic bands come from unsaturated fatty acids with a molecular structure, a single double bond originating from erucic (C22:1) and oleic (C18:1) acids, and multiple double bonds from linoleic (C18:2) and linolenic (C18:3) acids (Table 1). For comparison, the Raman spectrum of mineral oil F is shown, which clearly differs from the Raman spectrum of vegetable oil A and oil AF containing vegetable oil as a component. In the Raman spectra of mineral oil (Oil F), only bands originating from bonds of saturated bands of CH_3_ and CH_2_ groups can be observed to occur in the range of 3000–2800 cm^−1^ and at 1440 cm^−1^.

During the selection process of the bands to be analysed, good band separation with clearly defined integration limits was taken into account in order to determine the integral intensity of the I_1655_/I_1440_ band. Due to the fact that the group of bands at 3008 cm^−1^ and 2840 cm^−1^ in the tested vegetable oil was not very characteristic, however, this band was not analysed.

The interpretation of the Raman spectra consisted in identifying the bonds originating in the bands of unsaturated bonds. On this basis, the integral intensity of the _I1655_/I_1440_ bands was determined as the value of the integral corresponding to the area under the peak.

**Figure 2:**
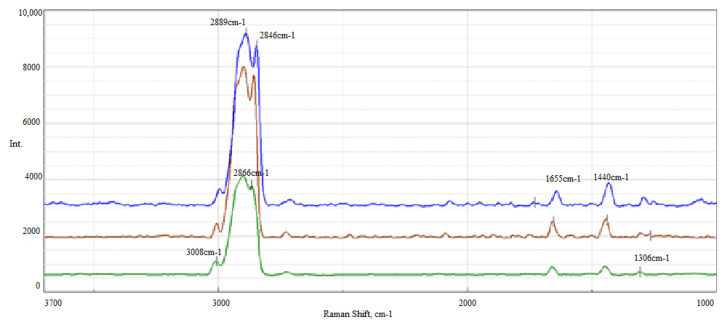
Raman spectra of A oil—green line, A_120 oil, oxidised at 120 °C—brown line and A_G_oz_ oil after anti-wear test—blue line, in the 3700–1000 cm^−1^ range.

The obtained Raman spectra formed the basis for the assessment of changes in the integral intensity of the I_1655_/I_1440_ bands of the tested vegetable lubricants. The basis for the assessment was the occurrence of multiple bond bands in vegetable oil, which allowed the examination of the changes occurring in the range of bands located at approximately 1655 cm^−1^ and 1440 cm^−1^ under the influence of destructive factors (Figure 2).

The greatest changes were observed for vegetable oil A after thermal oxidation carried out at 120 °C. The double bond vibration band at 1655 cm^−1^ after thermo-oxidation at 120 °C significantly decreased compared to oil A prior to the tests. The decrease in the integral intensity of the I_1655_/I_1440_ bands at the level of 79% is probably related to the oxidation of multiple bonds caused by the simultaneous action of oxygen and temperature during the PetroOxy test. Similar changes were observed after an hour-long test, but at a lower level of approximately 32% (Table 2).

**Figure 3:**
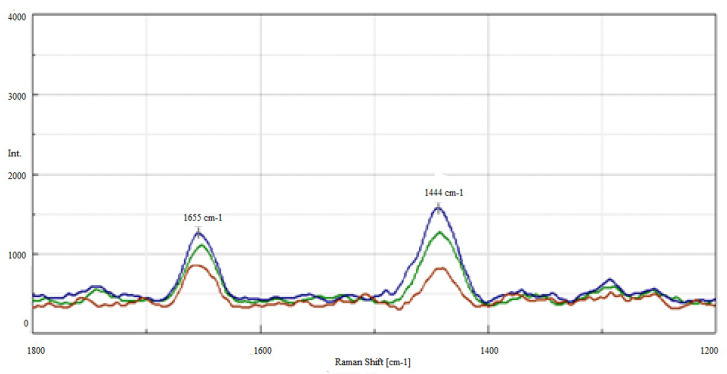
Raman spectra of A grease—green line, A_120 grease, oxidised at 120 °C—brown line and A_G_oz_ grease after anti-wear test—blue line, in the 1800–1200 cm^−1^ range.

In the Raman spectrum of oil A, there are no bands originating in carbonyl groups (Figure 3). In the conducted tests, however, the process of oxidation of Abyssinian oil (oil A) is accompanied by changes in the structure of fatty acid chains, and a slight increase in intensity within the band in the range of 1700–1760 cm^−1^ derived from carbonyl groups. This may indicate the processes of oxidation and thermal degradation of fatty acids to ketones and aldehydes. Depending on the type of excitation, a shift of bands associated with carbonyl groups is observed.

**Figure 4:**
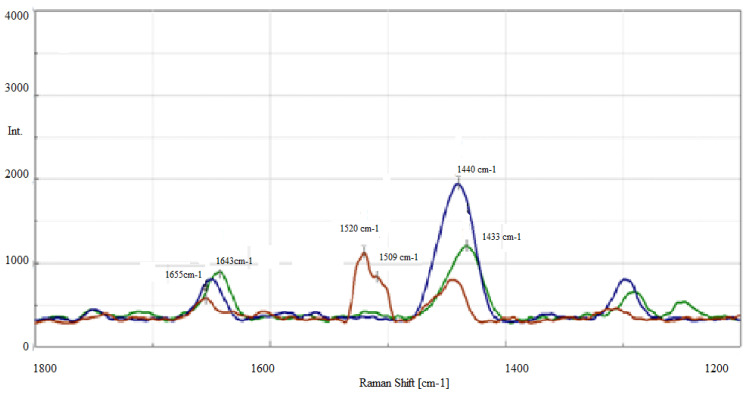
Raman spectra of AF grease—green line, AF_120 grease, oxidised at 120 °C—brown line and AF_G_oz_ grease after anti-wear test—blue line, in the 1800–1200 cm^−1^ range.

The Raman spectra obtained for grease A and grease AF before and after thermal oxidation carried out at a temperature of 120 °C (grease A_120, grease AF_120) and after an hour-long wear test (grease A_G_oz_, grease AF_G_oz_) are shown in Figure 3 and Figure 4.

The research shows that the spectra of the agents after the tests were significantly different. In the case of grease A_120, a substantial increase in the double bond vibration band located at 1655 cm^−1^ was observed. The intensity of this band could be affected by compounds with conjugated double bonds formed during heating. It is difficult to explain the mechanism of and reason behind the increase in double bonds in the case of vegetable grease A_120 after thermo-oxidation. It is possible that the mechanism of degradation of the oxidised polymer formed after the oligomerisation of fatty acids competes with the oxidation reaction, hence the observed increase in the integral intensity of the I_1655_/I_1440_ bands.

Conversely, for the A_G_oz_ grease, a slight decrease in the surface area under the peak of this band was observed after an hour-long anti-wear test, which resulted in a slight change (approximately 4%) of the integral intensity of the I_1655_/I_1440_ bands. The extortions carried out in this test slightly changed the degree of grease unsaturation, which may indicate the resistance of grease structure A to mechanical excitation.

For grease AF, the double bond vibration band at 1655 cm^−1^ both after thermo-oxidation (A_120) and after the one-hour test (A_G_oz_) decreased compared to the grease before the tests. Compared to that, in the spectra of the AF_120 grease after thermal oxidation, a clear reduction of the bands coming from the vibrations of the double bond at 1655 cm^−1^ and 3008 cm^−1^, and of the methylene groups at 1440 and 2850 cm^−1^ was observed. In the spectra of the grease AF after thermal and mechanical excitation, a reduction in the vibration intensity of the band characteristic for groups of polyunsaturated fatty acids at 1655 cm^−1^ was observed.

**Figure 5:**
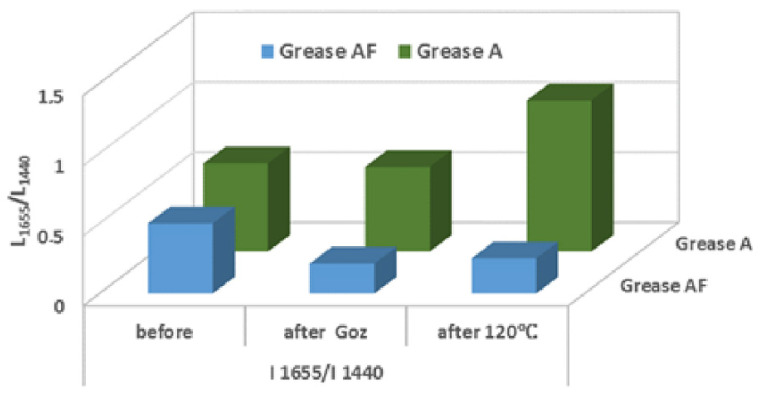
The influence of thermo-oxidation (Petrooxy) and mechanical forces (G_oz_) on the change of ratio of intensity integral unsaturated bonds C=C to the saturated bonds C-C of greases: vegetable Grease A and vegetable-mineral Grease AF.

The areas under the peak of the I_1655_/I_1440_ bands adopted for analysis were calculated, in order to reveal the characteristic signals indicating the progressive degradation of lubricants. The obtained results, depending on the type of excitation, are shown in Figure 5.

The observed diversification of the Raman spectra obtained for the lubricants in question after applying the inputs in the tests was related to the testing conditions (temperature, pressure, oxygen access, load). This indicates the different nature of changes taking place in the analysed measures. The changes found are the result of transforming the ingredients during the tests. The degree of unsaturation of polyene acids changed in the tested lubricants.

Through Raman spectroscopy, a change in the bands from unsaturated bonds in vegetable oil fatty acids was observed under the influence of both thermo-oxidation and the load of the friction node in the hour-long anti-wear test. Analysing a set of such bands, originating in unsaturated and saturated bonds present in the fatty acids of vegetable oil, the change in the degree of unsaturation was determined by comparing the intensity ratio of the bands before and after the tests. It was found that Raman spectroscopy allows the monitoring of changes occurring under the influence of thermal and mechanical excitation in the chemical structure of vegetable lubricants, which is closely related to the degree of unsaturation of the vegetable oil base. The ratio of the area under the peak of the bands of unsaturated and saturated bonds, derived from fatty acids contained in vegetable oil, was adopted as the criterion for the degree of unsaturation. The interpretation of the Raman spectra made it possible to assess the changes occurring in the chemical structure of vegetable grease following excitation.

The identified bands (I_1655_/I_1438_) present in the Raman spectrum of the tested vegetable lubricants, characterising the degree of unsaturation of fatty acids, were the basis for comparison with the resistance to oxidation (T_ON_), a parameter determined by the DSC method.

**Figure 6:**
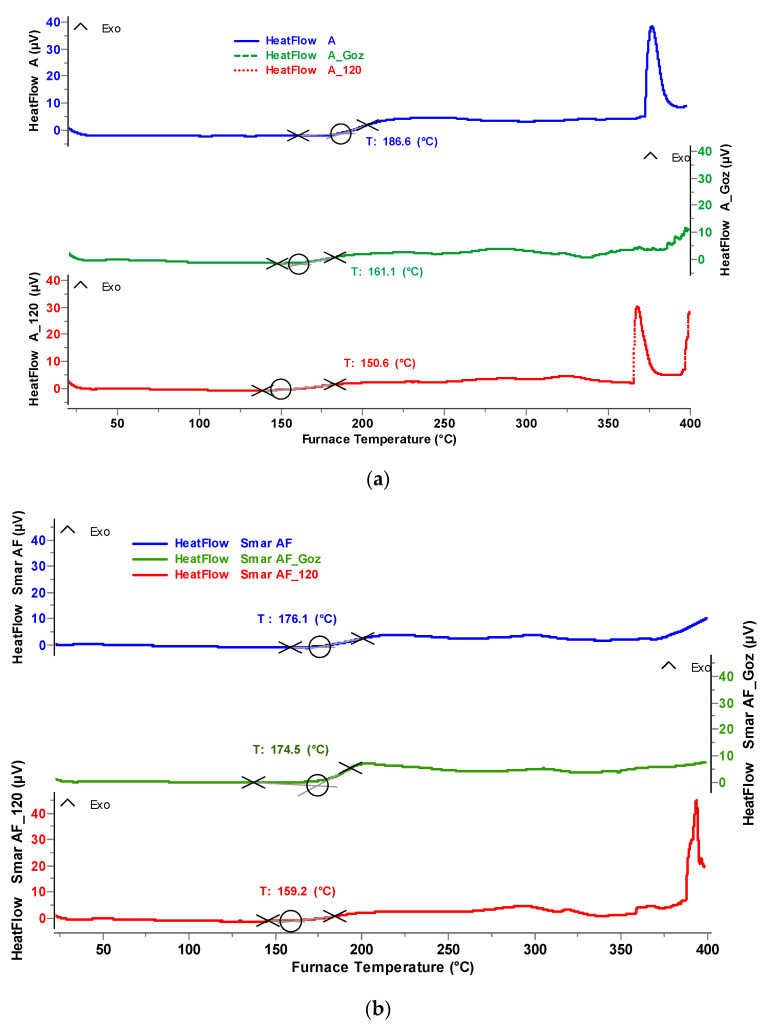
The change of resistance to oxidation after thermo-oxidation and mechanical forces for: (**a**) vegetable grease A, (**b**) vegetable–mineral grease AF.

The influence of thermo-oxidation (120 °C) and mechanical and thermal forces (G_oz_) on the change of resistance to oxidation of grease (A, AF) was evaluated based on the DSC (Differential Scanning Calorimetry) method (Figure 6).

As can be seen from the graph above, vegetable grease A is stable up to a temperature of approximately 186 °C, at which a slight exothermic effect occurs, which indicates the start of the oxidation process of grease A. In the case of vegetable–mineral grease AF, the oxidation process starts at a slightly lower temperature of about 176 °C. The observed exothermic effects occur at different temperatures, which may indicate different processes. It can be assumed that at the initial temperature, only those oxidation processes that require the lowest energy expenditure take place. At temperatures above 300 °C, however, in addition to oxidation and oligomerisation reactions, fatty acid degradation reactions may occur, characterised by a clear exothermic effect. The greatest decrease in resistance to oxidation was observed in the case of vegetable grease A, both after thermal and mechanical forcing (Table 3). Much less intense changes were observed for the AF grease, which can be explained by the beneficial effect of the plant–mineral oil base with increased resistance to oxidation.

**Figure 7:**
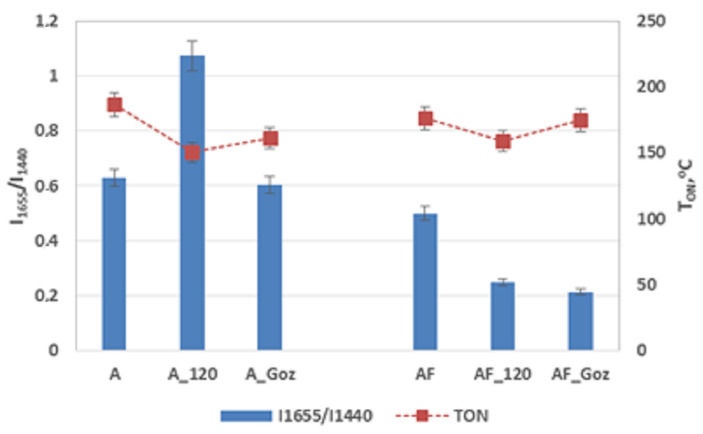
The change of resistance to oxidation and the change of ratio of the intensity integral of unsaturated bonds C=C to saturated bonds C-C after thermo-oxidation and mechanical forces for vegetable grease A and vegetable–mineral grease AF.

Under the influence of thermo-oxidation and tribosystem conditions (load, temperature and spindle speed) in the hour-long anti-wear test, there was a change in the bands derived from the unsaturated bonds of tested greases, which resulted in a reduction of the oxidation resistance of grease A (Figure 7).

It was found that there is a correlation between the ratio of the number of unsaturated to saturated bonds (I_1655_/I_1440_) and the resistance of vegetable lubricants to excitation. The decrease in the integral intensity of the bands (I_1655_/I_1440_) indicates a change in the chemical structure of vegetable lubricants under the influence of the forces applied in the tests. The obtained results confirmed that the changes taking placehave a significant impact on the resistance of vegetable lubricants to the oxidation process, although this is not a linear relationship, and the change depends on the content of vegetable oil in the tested lubricants.

## 3. Experimental Methods

Lubricants developed on the basis of vegetable and vegetable–mineral oils were tested. The following base oils were used: non-edible *Crambe Abyssinic* vegetable oil (oil A), pharmaceutical grade Finavesta A360B deeply refined mineral oil (oil F), and a mixture of vegetable and mineral oils (oil AF). A mineral oil of viscosity grade ISO VG 68 was mixed with vegetable oil A of viscosity grade ISO VG 46 in a ratio of 1:1.7. On the basis of these oils, vegetable grease A and vegetable–mineral grease AF were produced using an inorganic nano-silica thickener [32,33]. Then, the lubricants were subjected to thermal and mechanical forces. Following the tests, the samples were marked: after thermal oxidation, A_120_ grease, AF_120_ grease, and after an hour-long wear test, A_Goz_ grease, AF_Goz_ grease.

The characteristics of the base oils constituting the dispersion phase of the developed lubricants are presented in Table 4. The viscosity–temperature properties of the starting oils were assessed on the basis of kinematic viscosity determined at 40 °C and 100 °C in accordance with the PN-EN ISO 3104:2004 standard, and the viscosity index in accordance with the PN-ISO 2909:2009 standard. The lubricating properties, oxidation stability and resistance to the oxidation process were determined for the base oils and greases developed on these bases. Table 5 presents the properties of the developed lubricants based on vegetable oil and the mixed vegetable–mineral oil base.

The lubricating properties were determined in an hour-long anti-wear test. The tests were carried out under conditions of constant load of the friction node (392 N) and constant rotational speed of the spindle (500 ± 20 rpm). The duration of the test was one hour. The tested balls are made of 100Cr steel. The measure of wear resistance was the average diameter of flaws formed on the surfaces of the balls (d) and the limit wear load G_oz_. The G_oz_ parameter was calculated from the formula: G_oz_ = 0.52 × (P/d^2^), where: d—mean diameter of the friction track in mm, P—applied load of 392 N. The diameter of the wear track is reduced, i.e., wear limit values increase.

Oxidative stability was determined in accelerated oxidation tests using the PetroOxy^TM^ apparatus by PetroTest Instrument. The lubricants were oxidised at 120 °C, while maintaining the same sample weight of 10 ± 0.1 g. The measure of oxidative stability was the oxidation induction time (t_OI_) determined as the time needed to obtain a change in the maximum oxygen pressure by 10%.

The resistance to oxidation of base oils and lubricants was determined using differential scanning calorimetry (DSC) by LABSystem SETARAM TG/DTA/DSC. The experiments were carried out in dynamic conditions, in a temperature range from 20 °C to 400 °C, with a linear increase in temperature (5 °C/min), in an oxidising atmosphere, with a constant rate of oxygen flow in the measuring chamber (60 mL/min).

The Raman spectra were obtained using an NRS 5100 confocal grating Raman microspectrometer (Jasco Corporation, Tokyo, Japan). The research was carried out using a laser with a wavelength of 532.12 nm and a CCD detector. Spectrometer operating parameters: diffraction grating 1800 lines/mm, laser power 5.4 mW, numerical aperture 4000 µm, resolution 4.2 cm^−1^, objective magnification 100×, exposure time 15 s.

An inorganic thickener, Aerosil 300 fumed silica with hygroscopic properties, composed of microscopic spherical particles (7 nm) and a very large specific surface (270–330 mg^2^/g), was used to create the lubricants.

**Figure 8:**
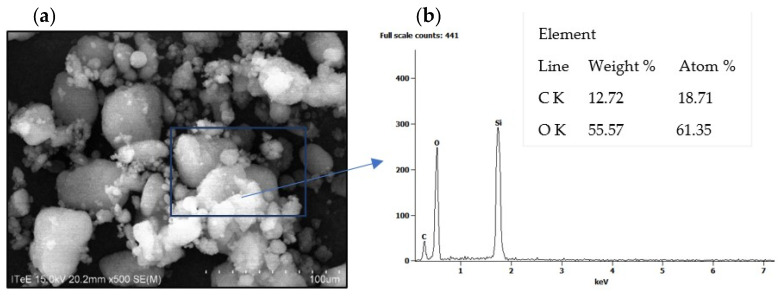
The Aerosil 300 silica thickener (**a**) SEM image over ×500 and (**b**) EDS spectrum with elemental analysis.

The SEM/EDS tests of the Aerosil thickener confirmed its high purity and the atom-only content of the molecule (Figure 8). In the EDS X-ray spectra from the surface of the thickener particles, intense signals indicating the presence of silicon and oxygen in the particle were identified. The presence of carbon in the EDS spectrum comes from the double-sided carbon tape on which the sample was attached.

Siloxane (Si-O-Si) and silanol (-Si-OH) groups were found on the surface of Aerosil. Hydrogen bridges were formed between the silanol groups of adjacent Aerosil particles, thanks to which spatial structure is formed in liquids. This property has been used to produce vegetable silica greases (Grease A, Grease AF) and mineral grease (AFIN grease) [32,33]. The prepared greases contained 8% (*m*/*m*) thickener. The basic properties of the developed lubricants obtained with the use of the silica thickener are presented in Table 5.

The research material consisted of samples of lubricants (grease A, grease AF) following tests on oxidation stability (grease A_120, grease AF_120) and lubricating properties (grease A_G_OZ_, grease AF_G_OZ_). Raman spectrophotometry and differential scanning calorimetry (DSC) were used to assess the changes occurring under the influence of thermal and mechanical excitation. Measurements were carried out before and after thermal oxidation at 120 °C and after an hour-long anti-wear test.

## 4. Conclusions

The usefulness of Raman spectroscopy as an analysis method to assess the impact of the type of components on structural changes between lubricants based on vegetable and vegetable–mineral oil was demonstrated. Raman spectroscopy allowed to monitor changes taking place under the influence of thermal and mechanical excitation in the chemical structure of bio-lubricants. Raman spectroscopy in conjunction with DSC analysis was used to assess changes in the degree of unsaturation and resistance to oxidation of vegetable lubricants under the influence of thermal and mechanical forces.

A new approach to the lubrication of tribological systems based on the use of spectroscopic methods can contribute to the identification and explanation of changes occurring under the influence of excitation in the structure of the lubricant, affecting performance properties and in particular resistance to oxidation.

## 5. Patents

The research descibed above is the subject of two patent applications concerning greases entitled “Highly specialized lubricant intended for use in agricultural and food industry machines and devices” by Drabik, J.; Kozdrach, R.; Wolszczak, M. Patent No 238661 which were received 4 October 2018 and „Grease, especially for lubricating machinery and equipment in the food industry” by Drabik, J.; Kozdrach, R.; P.427319, which was submitted on 2 October 2018 [in Polish].

## Figures and Tables

**Table 1:** The chemical shifts and vibration modes present in the Raman spectra of vegetable oil.

Raman Shift, cm^−1^	Bond	Vibration Mode
3100–2800	=C-H, C-H	stretching
1655	C=C	stretching
1440	C-H	stretching
1306	-C-H	scissoring
1266	=C-H	twisting

**Table 2:** The impact of excitation on the integral intensity of I_1655_/I_1440_ bands.

Lubricants	I_1655_/I_1440_	I_1655_/I_1440_ after PetrOxy at 120 °C	% Change I_1655_/I_1440_ after PetrOxy at 120 °C	I_1655_/I_1440_ after Goz	% Change I_1655_/I_1440_ after Goz
Oil					
Oil A	0.9315	0.1919	−79.4	0.6344	−31.9
Grease					
Grease A	0.6289	1.0739	+70.7	0.6039	−3.97
Grease AF	0.5001	0.2495	−50.1	0.2128	−57.4

**Table 3:** The impact of extortions on changing resistance to the lubricant oxidation process—DSC method.

Bio-Grease	Oxidative Resistance T_ON_, °C	Oxidative Resistance after Petrooxy 120 °C T_ON_, °C	% Change T_ON_, after Petrooxy 120 °C	Oxidative Resistance after G_oz_T_ON_, °C	% Change T_ON_ after G_oz_
Grease A	186.6 ± 4.2	150.6 ± 2.6	−19.5	161.1 ± 2.7	−14.3
Grease AF	176.1 ± 3.1	159.2 ± 2.2	−9.6	174.5 ± 2.3	−0.9

**Table 4:** The characteristics of base oils: vegetable A, vegetable–mineral AF, mineral F.

Oil	Onset Temp. of Oxidation T_ON_, °C	Oxidation Induction Time at 120 °C t_OI_, h	Lubricating Properties		Kinematic Viscosity, mm^2^/s		Viscosity	
			G_oz_ N/mm^2^	d, mm	40 °C	100 °C	ISO VG	VL
Oil A	175 ± 3.1	2.58 ± 0.12	586.2 ± 22.3	0.59 ± 0.02	46.9 ± 0.8	10.1 ± 0.9	46	208
Oil AF	185 ± 3.0	2.21 ± 0.14	498.2 ± 14.3	0.64 ± 0.04	51.2 ± 1.2	9.56 ± 0.6	46/68	175
Oil F	190 ± 4.3	2.24 ± 0.17	964.3 ± 27.3	0.46 ± 0.02	69.9 ± 1.4	9.12 ± 0.7	68	105

**Table 5:** The physical and chemical properties of the following greases: vegetable A, vegetable–mineral AF and mineral AFIN.

Grease	Penetration NLGI/ Consistency Grade	Oxidation Induction Time at 120 °C; t_OI_, h	Lubricating Properties		Dropping Temperature °C	Resistance to the Oxidation Process, T_ON_, °C
			G_oz_ N/mm^2^	d, mm		
Grease A	267/2	1.82 ± 0.12	1275 ± 35	0.40 ± 0.02	244.5 ± 1.5	186.6 ± 4.2
Grease AF	357/0	2.54 ± 0.17	784 ± 22	0.51 ± 0.04	254.0 ± 2.0	176.1 ± 3.1
Grease AFIN	305/1/2	7.65 ± 0.24	699 ± 18	0.54 ± 0.02	228.5 ± 0.5	179.1 ± 2.5

## Data Availability

Not applicable.

## Sample Availability

Samples of the compounds are available from the authors.
